# Evaluation of *Candida* Species Colonization and Fungal Susceptibility Profile in the Oropharyngeal Mucosa of Patients Receiving Head and Neck Radiotherapy

**DOI:** 10.1155/tswj/9095472

**Published:** 2026-04-26

**Authors:** Arezoo Aghakouchakzadeh, Soheila Manifar, Zohreh Moghadam, Ahmadreza Mirzaei, Niayesh Daneshvarpour, Shima Afrasiabi

**Affiliations:** ^1^ Department of Oral and Maxillofacial Pathology, School of Dentistry, Alborz University of Medical Sciences, Karaj, Iran, abzums.ac.ir; ^2^ Department of Oral and Maxillofacial Medicine, School of Dentistry, Imam Khomeini Hospital Complex, Tehran University of Medical Sciences, Tehran, Iran, tums.ac.ir; ^3^ Faculty of Dentistry, Alborz University of Medical Sciences, Karaj, Iran, abzums.ac.ir; ^4^ Department of Dental Biomaterials, School of Dentistry, Tehran University of Medical Sciences, Tehran, Iran, tums.ac.ir; ^5^ Laser Research Center of Dentistry, Dentistry Research Institute, Tehran University of Medical Sciences, Tehran, Iran, tums.ac.ir

**Keywords:** antifungals, *Candida albicans*, head and neck cancer, nystatin, oral candidiasis, radiotherapy

## Abstract

Oral candidiasis is the most common opportunistic fungal infection in patients undergoing head and neck radiotherapy (RT). The present study is aimed at provide the best treatment for patients undergoing RT by determining the characteristics of the *Candida* species in these patients and investigating the antifungal sensitivity patterns against eight available drugs according to the Clinical and Laboratory Standards Institute (CLSI). In this descriptive cross‐sectional study, 30 patients underwent head and neck RT. All patients were examined in three stages using oral and pharyngeal mucus swabs, and nystatin was prescribed for patients with symptoms of candidiasis infection. Subsequently, clinical fungal specimens were investigated by direct microscopy and culture and tested for sensitivity to antifungal drugs. MIC_50_, MIC_90_, and geometric mean MIC were calculated for each drug. Evidence of fungal colonization was observed in 17 specimens at various stages, with *Candida albicans* being the most frequently identified species. All tested *Candida* isolates were sensitive to nystatin and amphotericin B, whereas *Trichosporon asahii* showed intrinsic resistance to echinocandins (caspofungin, anidulafungin, and micafungin), and *C. krusei* exhibited the expected intrinsic resistance to fluconazole. The observed sensitivity of *C. tropicalis* to the tested antifungal drugs is based on a single isolate and should be considered descriptive rather than generalizable. Cases of *C. albicans* strains showed moderate or dose‐dependent susceptibility to itraconazole, voriconazole, and caspofungin. The isolate of *T. asahii* was only sensitive to nystatin and amphotericin B. According to the laboratory and clinical results, nystatin is recommended for most patients. Finally, it is better to prescribe the antifungal drugs in each center according to their reported sensitivity.

## 1. Introduction

Head and neck cancer (HNC) consists of a group of different malignancies of the upper aerodigestive tract, which together represent the seventh most common type of cancer worldwide. The mortality and complications associated with these malignancies are very high [1]. With the advances in science and various treatment methods, the mortality rate from these malignancies has decreased, but unfortunately, in contrast to the increase in survival rate, more patients are living with the consequences of cancer treatment [2, 3]. Radiotherapy (RT) is one of the most common treatment methods used alone or in combination with other treatment methods such as surgery and chemotherapy. However, high doses of radiation can lead to various adverse reactions [4]. Due to mucosal atrophy and reduced regeneration of epithelial cells, patients undergoing RT suffer from oral mucositis [5]. Besides that, due to the altered amount of blood supply and fibrosis in the connective tissue and in the salivary glands, there is a reduction in saliva flow and consequently dry mouth [6]. Dry mouth and oral mucositis are two of the factors that put these people at high risk of oral infections, especially oral candidiasis [7]. Oral candidiasis can exacerbate the symptoms associated with oral mucositis and lead to a worsening of the patient′s clinical condition. Therefore, treatment of oral candidiasis is recommended if RT is planned in the HN region [8]. However, *Candida* species are found in 50% of the population as part of the oral microbiota and can become a pathogen even after the onset of RT [9]. Moreover, the increasing concern over non‐albican*s Candida* species and their differing susceptibility profiles has become an important clinical concern [10]. In addition, changes in the structure of the mucosal layer caused by oral candidiasis often allow bacteria and fungi to invade the damaged tissue and cause infection, increasing the risk of developing oral candidiasis [8]. Therefore, effective diagnosis and treatment are very important as the problems caused by cancer can lead to interruption and prolongation of RT, which in turn compromises cancer treatment and the patient′s recovery [11].

Oral topical treatments are recommended as the first line of treatment for moderate forms of oral candidiasis [12]. There is insufficient evidence to support the treatment of oral candidiasis with current treatment measures in cancer patients undergoing RT and further studies in this area are recommended. Recent studies have shown that the sensitivity of *Candida* species to antifungal drugs is decreasing [13]. Resistance to antifungal drugs has also been found in non‐albicans *Candida* species, which vary in geographical distribution [14]. Cases of antifungal resistance lead to ineffective treatments with increased dissatisfaction, mortality, and prolonged hospitalization [15]. This study provides insights into the oral colonization of *Candida* species in patients with HNC undergoing RT, a population at higher risk for fungal infections. It also reports the antifungal susceptibility patterns of *C. albicans* and descriptive data for non‐albicans *Candida* species to support the selection of effective and timely treatment.

## 2. Methods

### 2.1. Clinical Specimens and Sample Processing

The patients of this descriptive cross‐sectional study were examined at the Cancer Institute of Imam Khomeini Hospital Complex affiliated to Tehran University of Medical Sciences. The study protocol was approved by the research ethics committee of Alborz University of Medical Sciences (R.ABZUMS.REC.1398.222). Inclusion criteria for this study are patients with a clinical and histological diagnosis of HNC (tumors of the nasal cavity and paranasal sinuses, nasopharynx, oral cavity, oropharynx, larynx, hypopharynx, salivary glands, thyroid gland, eye, and ear). These patients had to undergo RT over 30 to 35 sessions with a daily dose of 2 Gy. Patients were excluded if they did not have HNC, if they had received a full course of RT with a dose of 40 Gy or less prior to the study, if they were receiving chemotherapy at the time of the study, or if they had received antifungal medication within 3 months prior to the start of the study. The samples were prepared in three stages using two sterile cotton swabs. The first stage took place one to 3 days before the start of RT, the second stage 2 weeks after the start of RT, and the third stage in the fourth week after the start of RT (end of RT). The samples were placed in test tubes containing 0.5 ml of sterile water and immediately transported to the laboratory in a carrier bag. At each stage, patients with a fungal infection were administered a 100000 *μ*L/mL nystatin suspension four times a day for a fortnight [16]. Samples were interpreted in the context of patients′ clinical symptoms to help distinguish infection from colonization.

### 2.2. Diagnosis and Culture

In order to identify the species, Sabouraud dextrose agar (Merck, Germany) with chloramphenicol (50 *μ*g/mL) was used. Incubation was carried out at 37°C for 20 h. The cultured colonies were transferred to CHROMagar culture medium (CHROMagar Co., France). After a 24 h incubation at 35°C, different *Candida* species were distinguished based on color, surface texture, and conventional yeast identification methods. For example, *C. albicans* produced green colonies, *C. tropicalis* produced blue colonies, and *Candida krusei* produced pink colonies. Further tests such as morphology on cornmeal‐agar with Tween 80 (Merck, Germany) and microscopic identification were performed [11, 17–19]. Differentiation between *Candida* colonization and infection was based on a combination of clinical signs (such as erythema, white patches, pain, or inflammation) and positive culture results, as morphological identification alone cannot reliably distinguish colonization from true infection. *Trichosporon* isolates were identified based on morphological characteristics and are reported as putative *T. asahii*.

### 2.3. Antifungal Susceptibility Testing (AFST)

In this study, all clinical isolates of *Candida* species were tested using the microdilution broth method to determine the minimum inhibitory concentrations (MICs) of antifungal drugs, according to the Clinical and Laboratory Standards Institute. For antifungal agents without official CLSI clinical breakpoints (e.g., nystatin, itraconazole for certain species, caspofungin, and anidulafungin), interpretation was based on epidemiological cutoff values (ECVs) as defined in CLSI M57S and related documents [20]. In the microdilution broth method, the MIC of the isolates was calculated for antifungal drugs such as fluconazole (Pfizer, Groton, Connecticut, United States), voriconazole (Pfizer), itraconazole (Pfizer), amphotericin B (Sigma), nystatin (Jaber‐Ebne‐Hayyan Pharmaceutical Company, Iran), caspofungin (Merck), micafungin (Merck), and anidulafungin (Merck). According to CSLI‐M27‐A3, RPMI 1640 culture medium with glutamine (Sigma) without sodium bicarbonate with pH indicator was used to dilute the drug stock and prepare the fungal suspension. These drugs were prepared as powder in specific concentrations in the desired solvents according to the formula. The solvent for amphotericin B, voriconazole, itraconazole, and anidulafungin was prepared in dimethyl sulfoxide and the solvent for fluconazole, nystatin, caspofungin, and micafungin is sterile‐distilled water. To prepare a medicinal working solution, 100 *μ*L medicinal stock was dissolved with 9.9 mL of RPMI. A fungal suspension was then prepared at a concentration of 5 × 10^3^ mL/cell and confirmed with the spectrophotometer (Unico UV‐2100). A volume of 100 *μ*L of the stock drug solutions with desired concentrations was placed in a round‐bottomed 96‐well plate and diluted with phosphate‐buffered saline. The wells were then inoculated with 100 *μ*L of the fungal strains. The microplates were incubated at 37°C for 24 h to determine the visible MIC. The results were compared with the control well without drug. The lowest drug concentration that caused a significant reduction in growth was used as MIC. Also, *Candida parapsilosis* strains (ATCC22019) and *C. krusei* (ATCC6258) were examined for quality control of the test [21, 22].

### 2.4. Disk Diffusion Method

The agar disk diffusion method was employed to ensure the relationship between disk diffusion inhibition zone diameters and MIC of nystatin. Briefly, a suspension of *C. albicans* (10^6^ cells/mL) was spread on the Mueller–Hinton agar supplemented with glucose and methylene blue. Paper disks were impregnated with nystatin (Himedia, India; 25 *μ*g/disk) and placed on the inoculated plates. These plates were incubated at 35°C for 24 h. The diameters of the inhibition zones were measured in millimeters. The recommended susceptibility zone diameter breakpoints chosen in this study were ≥ 25 mm (according to CLSI guidelines) [23].

### 2.5. Statistical Analysis

A descriptive analysis of patient demographics and clinical characterization was performed, and the data were entered into the SPSS software for data analysis. The *p* value < 0.05 was considered significant.

## 3. Results

### 3.1. Patients and Characteristics

In this study, after applying the inclusion criteria, 30 patients (10 women and 20 men) with a mean age of 52 years and an age range of 35–83 years with a definite diagnosis of HNC were included. Patient demographics are listed in Table 1. Squamous cell carcinoma (27/30, 90%) was the most common type of cancer, followed by adenoid cystic carcinoma (ACC, 1/30, 3.3%), lymphoma (1/30, 3.3%), and one patient with hemangioma, a benign vascular lesion (1/30, 3.3%). Of the participants in this study, seven people (23.3%) smoked and three (10%) consumed alcohol. Of the participants in this study, 21 people (70%) had a history of an underlying medical condition. The most common underlying conditions, in order of frequency, included diabetes in eight patients (26.7%), hypertension in five patients (16.7%), a history of cancer in three patients (10.0%), fatty liver in two patients (6.7%), hypothyroidism, and rheumatism in one person (3.3%).

**Table 1:** Demographic information of the studied population.

Variable	Group	*N*(%)
Gender	Male	20 (66/7)
	Female	10 (33/3)
Age	< 50 years	10 (33/3)
	> 50 years	20 (66/7)
Comorbidities	Yes	21 (70)
	No	9 (30)
Smoking	Smokers	7 (23.3)
	Nonsmokers	23 (76.6)
Alcohol	Using currently	3 (10)
	Never used	27 (90)
Cancer type	SCC	27 (90)
	ACC	1 (3/3)
	Lymphoma	1 (3/3)
	Hemangioma^a^	1 (3/3)
Cancer site	Oropharynx	27 (90)
	Other sites	3 (10)
Total		30 (100)

Abbreviations: ACC, adenoid cystic carcinoma; SCC, squamous cell carcinoma.

^a^Hemangioma (benign vascular lesion).

### 3.2. Incidence of Fungal Colonization

**Figure 1:**
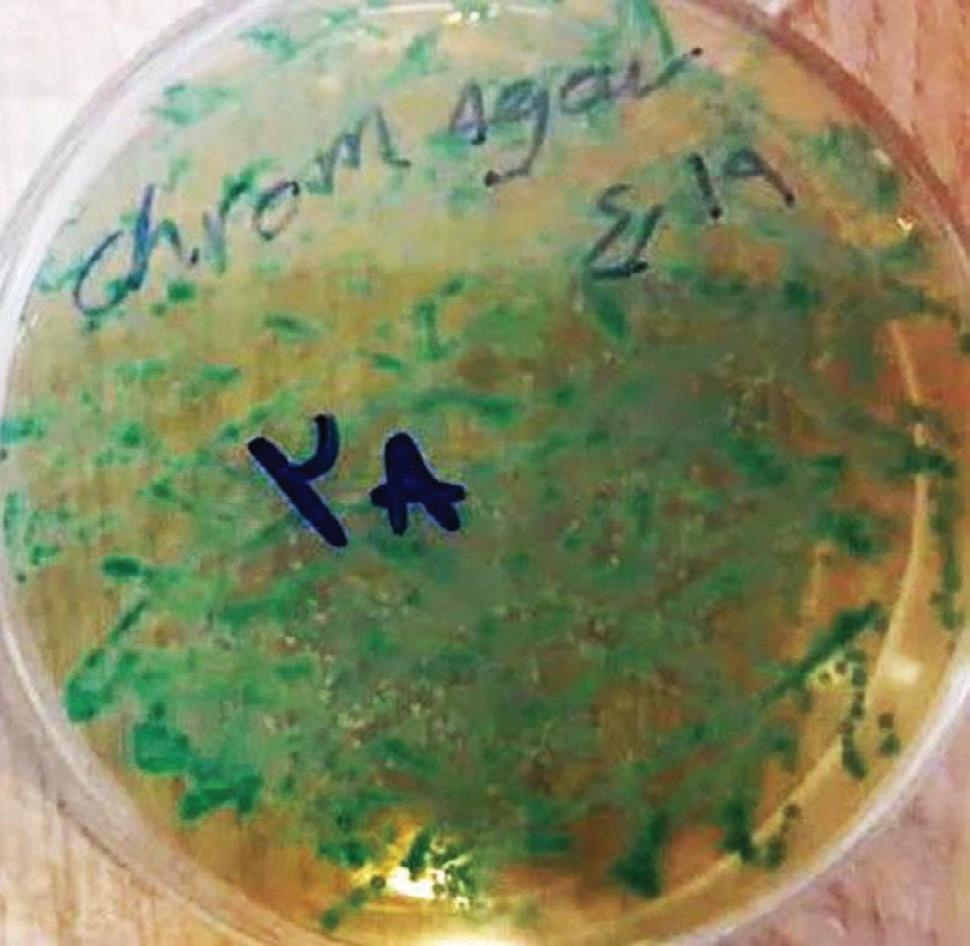
Representative *Candida albicans* colony on CHROMagar isolated in this study.

Of the 30 participants in this study, 14 showed symptoms of a fungal infection, seven patients already suffered from a fungal infection before the start of RT, which did not improve in two of them after 2 weeks of nystatin administration. In one patient, symptoms of fungal infection persisted until the end of the fourth week, despite the prescription of nystatin and its demonstrated efficacy in laboratory susceptibility testing. Shortly afterward, the patient died due to a critical general condition, and further treatment was not possible. In another person, the symptoms of fungal infection disappeared after the administration of nystatin for another 2 weeks. Among the participants, six of them showed symptoms of fungal infection at 2 weeks after receiving the RT dose, so the total number of infected individuals in the second week of RT was eight. After a 2‐week administration of nystatin to all of these eight patients, all recovered. In addition, one of the participants in this study (5.88%) showed symptoms of fungal infection at the end of the RT course (4 weeks). Therefore, in the fourth week, two of the total participants (6.66%) showed symptoms of fungal infection. The person newly infected in the fourth week of RT also recovered at the end of the RT course and without the radiation dose after 2 weeks of posttreatment. A total of 17 cases (56.66%) of fungal infections at different stages were observed. The most common fungal species identified was *C. albicans* with a frequency of 14 cases (82.35%). A representative *C. albicans* colony showing green coloration on chromogenic agar is presented in Figure 1. Other identified fungi that caused infection are listed in Table 2.

**Table 2:** Frequency of fungal species in all cultures.

*Candida* species	Oral *Candida* colonization no. (%)	Total no. (%)
*Candida albicans*	14 (82.35)	17 (56.66)
*Candida tropicalis*	1 (5.88)	
*Candida krusei*	1 (5.88)	
*Trichosporon asahii*	1 (5.88)	

### 3.3. AFST

**Figure 2:**
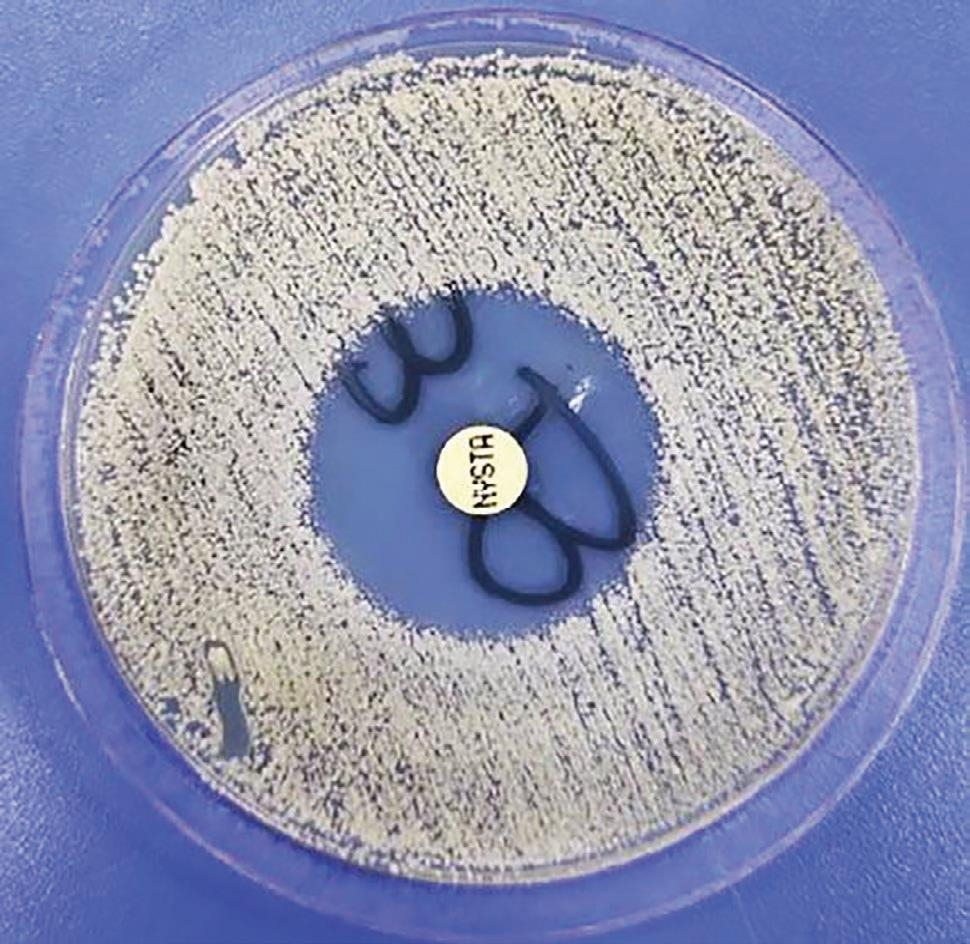
Agar disc diffusion assay of nystatin against *Candida* species.

The drug sensitivity of nystatin in 14 isolates of *C. albicans*, MIC_50_, and MIC_90_ were 0.312 *μ*g/mL and 0.625 *μ*g/mL, respectively. All 14 isolates (100%) were sensitive to nystatin. The drug sensitivity pattern of nystatin in *C. krusei* isolate, *C. tropicalis* isolate, and *T. asahii* isolate was also observed. Like nystatin, all isolates were sensitive to amphotericin B. All isolates of *C. albicans* were sensitive to anidulafungin, micafungin, and fluconazole. In addition, half of the isolates were dose‐dependently sensitive to itraconazole, and in some cases moderate sensitivity to voriconazole and caspofungin was seen. The isolates of *C. krusei* and *T. asahii* were not sensitive to fluconazole and the isolates of *C. tropicalis* were sensitive to this drug. The sensitivity pattern of itraconazole in isolates of *C. krusei* and *T. asahii* was dose‐dependent and sensitive in *C. tropicalis* isolates. Drug sensitivity pattern of voriconazole in *C. krusei* isolates and *C. tropicalis* isolates was sensitive and, in a *T. asahii* isolate, it was intermediate. The interpretation of the results can be found in Table 3. Furthermore, a drug sensitivity test was performed using the disk diffusion method for three isolates of *C. albicans*, one isolate of *C. krusei*, one isolate of *C. tropicalis*, and one isolate of *T. asahii*, and all isolates tested were sensitive to nystatin (Figure 2).

**Table 3:** Interpretation of drug susceptibility test.

*Candida* species	Antifungal agents	Range (*μ*g/mL)	MIC_50_ (*μ*g/mL)	MIC_90_ (*μ*g/mL)	GM	R%	I%	SDD%	S%
*Candida albicans*	Nystatin	0.019–10	0.312	0.625	0.496	—	—	—	100
	Fluconazole	0.062–64	0.5	2.0	0.602	—	—	—	100
	Itraconazole	0.032–16	0.125	0.5	0.189	—	—	50	50
	Voriconazole	0.032–16	0.062	0.25	0.082	—	14.3	—	85.7
	Amphotericin B	0.032–16	0.062	0.5	0.075	—	—	—	100
	Caspofungin	0.007–8	0.062	0.125	0.086	—	7.1	—	92.9
	Anidulafungin	0.007–8	0.031	0.125	0.034	—	—	—	100
	Micafungin	0.007–8	0.008	0.016	0.012	—	—	—	100
*Candida krusei*	Nystatin	0.019‐10	—	—	—	—	—	—	100
	Fluconazole	0.062–64	—	—	—	100	—	—	—
	Itraconazole	0.032–16	—	—	—	—	—	100	—
	Voriconazole	0.032–16	—	—	—	—	—	—	100
	Amphotericin B	0.032–16	—	—	—	—	—	—	100
	Caspofungin	0.007–8	—	—	—	100	—	—	—
	Anidulafungin	0.007–8	—	—	—	—	—	—	100
	Micafungin	0.007–8	—	—	—	—	—	—	100
*Candida tropicalis*	Nystatin	0.019–10	—	—	—	—	—	—	100
	Fluconazole	0.062–64	—	—	—	—	—	—	100
	Itraconazole	0.032–16	—	—	—	—	—	—	100
	Voriconazole	0.032–16	—	—	—	—	—	—	100
	Amphotericin B	0.032‐16	—	—	—	—	—	—	100
	Caspofungin	0.00–8	—	—	—	—	—	—	100
	Anidulafungin	0.007–8	—	—	—	—	—	—	100
	Micafungin	0.007–8	—	—	—	—	—	—	100
*Trichosporon asahii*	Nystatin	0.019–10	—	—	—	—	—	—	100
	Fluconazole	0.062–64	—	—	—	100	—	—	—
	Itraconazole	0.032–16	—	—	—	—	—	100	—
	Voriconazole	0.032–16	—	—	—	—	100	—	—
	Amphotericin B	0.032–16	—	—	—	—	—	—	100
	Caspofungin	0.007–8	—	—	—	100	—	—	—
	Anidulafungin	0.007–8	—	—	—	100	—	—	—
	Micafungin	0.007–8	—	—	—	100	—	—	—

Abbreviations: GM: geometric mean; I, intermediate; MIC, minimum inhibitory concentration, R, resistant; S, sensitive; SDD, susceptible‐dose dependent.

## 4. Discussion

Oral candidiasis is a common complication of RT in patients with HNC, especially in patients with oral cavity cancer [24]. In the present study, seven patients (23.3%) were found to have fungal colonization and symptoms prior to starting RT. Schelenz et al. reported the rate of oral candidiasis in cancer patients as 18.9%, which is consistent with our study [25]. In the study by Madiyal et al. [24] 79.0% of cancer patients were diagnosed with oral candidiasis within 4 weeks of RT (average 1.5–5 weeks). In another study, Dahiya et al. [26] reported that the prevalence of oral candidiasis in patients with HNC receiving RT treatment was 7.5% before, 37.5% during, and 32.5% after RT procedures. According to our study, most cases of oral candidiasis infections were observed 2 weeks after treatment. There are large differences in the epidemiology of oral candidiasis reported in different studies. These variations may be attributed to several factors, such as differences in cancer types, RT doses, demographic characteristics, and the difficulty in distinguishing candidiasis from mucosal reactions caused by RT. Other contributing factors include variations in risk factors for oral candidiasis (such as oral hygiene and predisposing colonization history) and differences in the timing and treatment of symptomatic patients during various phases of the studies [27].

In the present study, the most commonly identified fungal species was *C. albicans*. According to most studies, *C. albicans* is still the most common cause of fungal colonization [15, 25, 26]. In contrast, in studies by Singh et al. [9] and Raj et al. [28], non‐albicans *Candida* species were the most commonly isolated fungal species. Among these, *Candida glabrata*, *C. tropicalis*, and *C. krusei* were identified as the predominant species [25]. A possible reason for this contrasting trend in the increase of non‐albican*s Candida* species in some studies could be the higher sensitivity of current diagnostic techniques, which leads to better identification of non‐albicans *Candida* species than in the past [22]. Due to the limited sample size, the prevalence of non‐albicans *Candida* species was not comparable in the present study.

Considering the 2‐week course of prescribed nystatin, cure was achieved in most patients with oral candidiasis infection. There were also no signs of fungal lesions, either clinically or in the laboratory tests at the reexamination 2 weeks later. According to the AFST by microdilution and disk diffusion method, the reason for the lack of recovery in a small number of patients was the wrong way of taking the medication. In this context, Maheronnaghsh et al. [29] showed that topical nystatin had the best effect (98.65%) on *C. albicans* and non‐albicans *Candida* species in in vitro conditions. They recommended the topical use of nystatin for prevention in high‐risk patients and for treatment of the first stage of infection.

However, resistance of 1%–6% was reported in some studies [30]. In addition to nystatin, amphotericin B was effective in all species according to the results of AFST. In the present study, *C. tropicalis* was the only isolate among all isolates that was sensitive to all eight drugs used in the study. Although in recent years the resistance of this yeast to antifungal drugs has increased, it has attracted much attention. In particular, resistance to azoles, especially fluconazole, has been widely reported. In this context, the authors argue that the increase in worldwide consumption of this drug is one of the main reasons for this phenomenon. However, studies on the molecular mechanisms underlying this phenomenon are still needed.

On the other hand, in the present study, some of the *C. albicans* strains showed moderate or dose dependent sensitivity to itraconazole, voriconazole, and caspofungin. Perea et al. [22] believed that factors such as intrinsic resistance of *C. albicans*. and the substitution of *C. albicans* by a more resistant species such as *C. krusei* and *C. glabrata* may play a role in the development of azole resistance in *C. albicans* isolates. On the other hand, in the study by Paphitou et al. [31], *T. asahii* strains were more sensitive to azoles compared with amphotericin B*. C. krusei* isolate was also resistant to fluconazole due to inherent resistance. MIC values for *C. krusei* isolates, *C. tropicalis,* and *T. asahii* cannot be calculated as only a single isolate was involved in each case due to the limited sample size of the present study. It should be noted that the susceptibility results for non‐albicans *Candida* species and single isolates are descriptive and should be interpreted with caution, considering potential intrinsic resistance.

### 4.1. Limitations of the Study

One of the advantages of this study is the identification of *Candida* species in the oral cavity of patients with HNC treated with RT to determine a group of patients with a higher risk of infection and consequently take the next necessary measures [11]. One limitation of the present study is the lack of molecular confirmation for fungal species identification. Identification was based on conventional phenotypic and morphological methods, which, although widely accepted and routinely used in clinical mycology, may have lower discriminatory power compared with molecular techniques. Future studies incorporating standardized molecular identification methods are recommended to further validate these findings [32]. Morphological identification confirms the presence of *Candida* but alone cannot differentiate colonization from infection; this distinction should be made in conjunction with clinical presentation. The small sample size (30 patients, 17 positive cultures) and the physical weakness of these patients, which increases their susceptibility to this disease, are significant limitations. These factors limit the strength of epidemiological conclusions and make the observed susceptibility patterns for non‐albicans *Candida* species descriptive rather than definitive. It is suggested to conduct a new study with more samples to achieve optimal drug response and more accurate documentation, monitor epidemiological trends and evaluate drug sensitivity in different *Candida* species It is also better to determine the variables such as cancer type and location that play a role in the fungal colonization of cancer patients in order to obtain more accurate results. If drug resistance of fungal species is detected in the clinic and in the laboratory, the molecular mechanisms of the cause of resistance are investigated. Definitive species‐level identification of *Trichosporon* requires molecular methods such as PCR and sequencing [33].

## 5. Conclusion

Attention to clinical signs during oral examination could improve decision‐making in the detection of oral candidiasis. Full knowledge of the yeast species present in patients is a fundamental step to implement the instructions necessary for the best treatment selection. Diagnosis is still regularly made based on clinical findings; however, a fungal culture may be necessary and useful, especially in patients with identifiable risk factors. Considering the efficacy of the nystatin according to laboratory and clinical results, the use of this drug is recommended for most patients. Ultimately, it is better to prescribe appropriate antifungal drugs according to their reported sensitivity in each center. In this way, the uncontrolled use of drugs and, of course, the development of secondary and unwanted drug resistance can be prevented.

## Author Contributions

A.A.: conceptualization, methodology, writing—original draft. S.M.: data curation, formal analysis, writing—original draft. Z.M.: data curation, formal analysis, writing—original draft. A.M.: conceptualization, methodology, formal analysis. N.D.: writing—original draft. S.A.: investigation, writing—review and editing.

## Funding

No funding was received for this manuscript.

## Disclosure

All authors read and approved the final manuscript.

## Ethics Statement

This study was approved by the research ethics committee of Alborz University of Medical Sciences (R.ABZUMS.REC.1398.222).

## Conflicts of Interest

The authors declare no conflicts of interest.

## Data Availability

Data sharing is not applicable to this article as no datasets were generated or analyzed during the current study.
